# Conditional Mesenchymal Disruption of *Pkd1* Results in Osteopenia and Polycystic Kidney Disease

**DOI:** 10.1371/journal.pone.0046038

**Published:** 2012-09-21

**Authors:** Ni Qiu, Zhousheng Xiao, Li Cao, Valentin David, Leigh Darryl Quarles

**Affiliations:** Department of Medicine, University of Tennessee Health Science Center, Memphis, Tennessee, United States of America; Institut de Génomique Fonctionnelle de Lyon, France

## Abstract

Conditional deletion of *Pkd1* in osteoblasts using either *Osteocalcin(Oc)*-Cre or *Dmp1*-Cre results in defective osteoblast-mediated postnatal bone formation and osteopenia. *Pkd1* is also expressed in undifferentiated mesenchyme that gives rise to the osteoblast lineage. To examine the effects of Pkd1 on prenatal osteoblast development, we crossed *Pkd1* ^flox/flox^ and *Col1a1(3.6)*-Cre mice, which has been used to achieve selective inactivation of *Pkd1* earlier in the osteoblast lineage. Control *Pkd1* ^flox/flox^ and *Pkd1* ^flox/+^, heterozygous *Col1a1(3.6)*-Cre;*Pkd1* ^flox/+^ and *Pkd1* ^flox/null^, and homozygous *Col1a1(3.6)*-Cre;*Pkd1* ^flox/flox^ and *Col1a1(3.6)*-Cre;*Pkd1* ^flox/null^ mice were analyzed at ages ranging from E14.5 to 8-weeks-old. Newborn *Col1a1(3.6)*-Cre;*Pkd1* ^flox/null^ mice exhibited defective skeletogenesis in association with a greater reduction in *Pkd1* expression in bone. Conditional *Col1a1(3.6)*-Cre;*Pkd1* ^flox/+^ and *Col1a1(3.6)*-Cre;*Pkd1* ^flox/flox^ mice displayed a gene dose-dependent decrease in bone formation and increase in marrow fat at 6 weeks of age. Bone marrow stromal cell and primary osteoblast cultures from homozygous *Col1a1(3.6)*-Cre;*Pkd1* ^flox/flox^ mice showed increased proliferation, impaired osteoblast development and enhanced adipogenesis *ex vivo*. Unexpectedly, we found evidence for *Col1a1(3.6)*-Cre mediated deletion of *Pkd1* in extraskeletal tissues in *Col1a1(3.6)*-Cre;*Pkd1* ^flox/flox^ mice. Deletion of *Pkd1* in mesenchymal precursors resulted in pancreatic and renal, but not hepatic, cyst formation. The non-lethality of *Col1a1(3.6)*-Cre;*Pkd1* ^flox/flox^ mice establishes a new model to study abnormalities in bone development and cyst formation in pancreas and kidney caused by *Pkd1* gene inactivation.

## Introduction

Polycystin-1 (PC1), the *PKD1* gene product, is a highly conserved, multi-domain membrane protein widely expressed in various cell types and tissues [1], [2]. The specific biological functions of PC1 (PKD1) in different tissues are currently being elucidated; best understood are the kidney functions. In renal epithelium, PKD1 forms a complex with the calcium channel, PKD2 that co-localizes to primary cilia and functions as a flow sensor regulating cell proliferation and polarity. Loss of PKD1 or PKD2 function in renal tubular epithelial cells causes Autosomal Dominant Polycystic Kidney Disease (ADPKD) [3], [4] that is the result of abnormal cell proliferation and cell polarity and leads to cystic kidney disease. *PKD1* mutations also lead to cystic disease of liver and pancreas in some patients with ADPKD, also because of abnormal proliferation of ductal epithelial cells in these tissues [5], [6], [7]. PKD1 may also have a role in the development and function in endothelial- and mesenchymal-derived cells. *PKD1* mutations lead to vascular (intracranial and aortic aneurysms) [8], [9], [10], [11] and lung (bronchiectasis) abnormalities [12]. PKD1 and PKD2 are required for placental development [13]. *Pkd1* mutations in mouse models also cause abnormalities of the skeleton [14], [15], [16], [17], [18] and human subjects with polycystic kidney disease appear to have earlier elevation of the bone-derived hormone FGF23 [19].

It has been difficult to determine the specific extra-renal functions of Pkd1/PKD1using mutations of *Pkd1/PKD1* in mice and humans. Global ablation of *Pkd1* in mice leads to a complex, embryonically lethal, phenotype [7]. Multiple abnormalities, including renal and pancreatic cysts and pulmonary hyperplasia, are observed in *Pkd1* ^−/−^ mice when they survive to embryonic day 15.5 post coitum (E15.5). In global knockout mice it is difficult to differentiate between indirect extra-renal abnormalities due to the effects of the complex metabolic alterations caused by renal cystic disease from direct effects caused by loss of Pkd1 functions in affected tissues. In addition, in humans, ADPKD is a heterozygous state, whereby mutations leading to loss of one *PKD1* or *PKD2* allele is combined with somatic mutations in the kidney (i.e., a second hit) to cause renal cystic disease [20], [21], [22], [23], [24]. The resulting residual function of the non-mutated *PKD1* or *PKD2* allele in extra-renal tissues may also mask discovery of PKD1 or PKD2 functions in non-renal tissues.

The *Pkd1* ^flox/flox^ mouse model has been used to define the tissue selective function of Pkd1 *in vivo* [25]. A low frequency of renal *Pkd1* gene inactivation and only a few renal cysts and more frequent hepatic cysts is reported from the conditional deletion of *Pkd1* in *MMTV-Cre* mice [25], whereas the broadly expressed tamoxifen-Cre inducible inactivation of the *Pkd1* gene in mice resulted in massive cystic transformation of renal tissue [26]. The selective deletion of *Pkd1* in kidney by using *Ksp*-Cre, or more broadly *Nestin*-Cre, also leads to the formation of polycystic kidneys resembling human ADPKD [27], [28].

In addition, use of *Pkd1* ^flox/flox^ mice and bone-specific Cre mice has defined previously unrecognized functions of polycystin-1 in bone. In this regard, the selective deletion of *Pkd1* in osteoblasts by using *Osteocalcin(Oc)*-Cre and in osteocytes by using *Dmp1*-Cre results in osteopenia in adult mice because of defects in osteoblast-mediated bone formation [17], [18]. At present, however, it is unclear whether the functions of Pkd1 are limited to mature osteoblasts and osteocytes or involve earlier stages in osteoblast development.

To explore the effects of Pkd1 on early pre-osteoblast stage and prenatal bone development, we used the *Col1a1(3.6)* promoter to drive Cre-recombinase expression (*Col1a1(3.6)*-Cre) in mesenchymal precursors. *Col1a1(3.6)* promoter driven Cre expression begins at E10 and peaks between E12.5 and E14.5 in developing skeletal elements [29], [30]. *Col1a1(3.6)*-Cre is thought to be specific for the osteoblast lineage and it has been extensively used to conditionally delete genes early in osteoblastic development and to study bone-specific function of many genes [29], [30], [31], [32].

We found that conditional deletion of *Pkd1* from osteoblasts precursors within the mesenchymal lineage resulted in defective bone formation that was associated with abnormal osteoblastic development and enhanced adipogenesis. Unexpectedly, we found that *Col1a1(3.6)*-Cre was not bone specific and resulted in deletion of *Pkd1* in multiple tissues, leading to cyst formation in the kidney and pancreas, but not the liver of adult mice.

## Results

### *Col1a1 (3.6)*-Cre-mediated conditional deletion of *Pkd1* in different tissues

**Figure 1:**
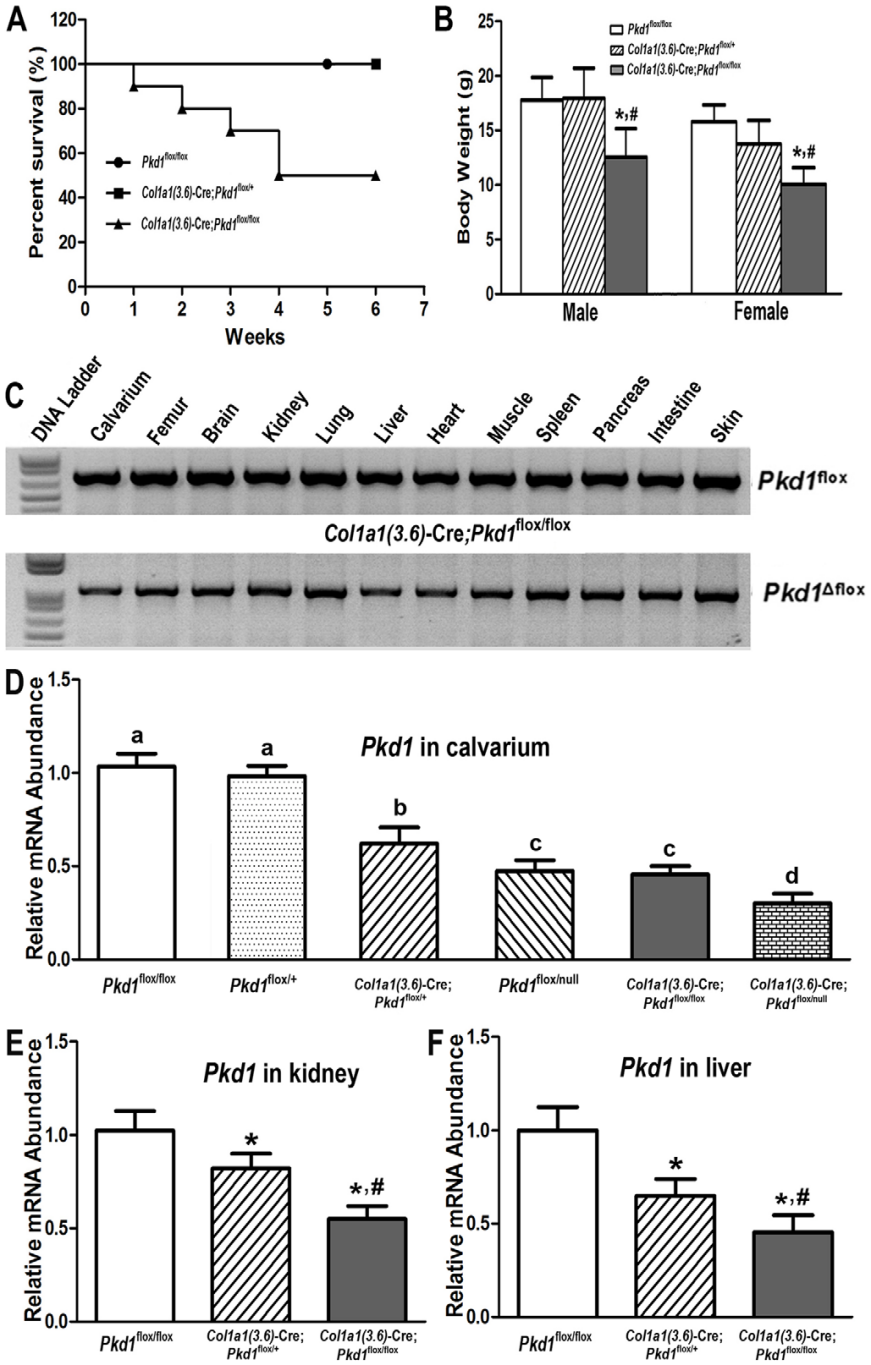
*Col1a1(3.6)*-Cre-mediated conditional deletion of *Pkd1* from the floxed *Pkd1* allele (*Pkd1* ^flox^) in different tissues. (A) Kaplan–Meier survival curve. Heterozygous *Col1a1(3.6)*-Cre; *Pkd1* ^flox/+^ had normal survival identical to control (*Pkd1* ^flox/flox^ ) mice, homozygous *Col1a1(3.6)*-Cre; *Pkd1* ^flox/flox^ mice began to die 1 week after birth and only half of these mice survived beyond 6 weeks. (B) Body weight of *Col1a1(3.6)*-Cre;*Pkd1* ^flox/+^ and control (*Pkd1* ^flox/flox^) mice were not different, but the body weight of both male and female *Col1a1(3.6)*-Cre;*Pkd1* ^flox/flox^ mice was reduced by ∼30% and ∼36% compared with the other two genotypes. (C) Genotyping PCR analysis of different tissues harvested from 6-week-old homozygous *Col1a1(3.6)*-Cre; *Pkd1* ^flox/flox^ mice showed that both *Pkd1* ^flox^ and *Pkd1* ^Δflox^ alleles existed in all tested tissues including bone and nonskeletal tissues, indicating that *Col1a1(3.6)*-Cre promoter is not specific for bone. (D–E) Real-time RT-PCR analysis of total *Pkd1* transcripts in calvaria from both *Col1a1(3.6)*-Cre;*Pkd1* ^flox/flox^ and *Col1a1(3.6)*-Cre;*Pkd1* ^flox/null^ models, and in kidney and liver from *Col1a1(3.6)*-Cre;*Pkd1* ^flox/flox^ model at 6 weeks of age. Total *Pkd1* transcripts were expressed as the fold changes relative to the housekeeping gene *β-actin* subsequently normalized to control *Pkd1* ^flox/flox^ or *Pkd1* ^flox/+^ mice. Data represent the mean ± SD from five or six individual mice. Values sharing the same superscript are not significantly different at *P*<0.05. *Significant difference from control (*Pkd1* ^flox/flox^); ^#^significant difference from heterozygous *Col1a1(3.6)*-Cre;*Pkd1* ^flox/+^ mice at *P<*0.05, respectively.

The four genotypes from the chosen breeding strategy (*Col1a1(3.6)*-Cre;*Pkd1* ^flox/flox^, *Col1a1(3.6)*-Cre; *Pkd1* ^flox/+^, *Pkd1* ^flox/flox^, and *Pkd1* ^flox/+^) were born at the expected Mendelian frequency. We investigated *Col1a1(3.6)*-Cre; *Pkd1* ^flox/flox^ (*Pkd1^Col1a1(3.6)-^* ^cKO)^, heterozygous *Col1a1(3.6)*-Cre;*Pkd1* ^flox/+^, and *Pkd1* ^flox/flox^ mice. *Pkd1* ^flox/flox^ was used as the control group. *Col1a1(3.6)*-Cre; *Pkd1* ^flox/+^ mice exhibited normal survival indistinguishable from control mice (*Pkd1* ^flox/flox^), whereas *Pkd1^Col1a1(3.6)^* ^-cKO^ mice displayed a 50% mortality rate in association with development of multiple cysts in the kidney and pancreas from newborn to 6-week-old mice (Fig. 1A). We observed no differences in body weight between heterozygous *Col1a1(3.6)*-Cre; *Pkd1* ^flox/+^ and control *Pkd1* ^flox/flox^ littermates; however , *Pkd1^Col1a1(3.6)-^* ^cKO^ mice were smaller and had a significantly lower body weight compared with *Col1a1(3.6)*-Cre;*Pkd1* ^flox/+^ and *Pkd1* ^flox/flox^ littermates at 6 weeks-of-age (Fig. 1B). Using an alternative breeding strategy with *Pkd* ^flox/null^ mice, we also generated *Col1a1(3.6)*-Cre;*Pkd1* ^flox/null^ mice, which had greater perinatal mortality that prevented collection of adult animals for analysis (data not shown). Therefore, the analysis of the skeletal phenotype of *Col1a1(3.6)*-Cre;*Pkd1* ^flox/null^ mice is limited to newborn mice.

*Col1a1(3.6)*-Cre-mediated excision occurs in the developing mesenchymal tissues between E12.5 and E14.5 [29], [30] and would be expected to carry forward into tissues developed from mesenchymal precursors. To determine the tissue distribution of *Col1a1(3.6)*-Cre-mediated deletion of *Pkd1* in adult mice, we performed PCR analysis in different tissues by using a combination of primers that specifically detect floxed *Pkd1* alleles (*Pkd1* ^flox)^ and the excised floxed *Pkd1* alleles (*Pkd1* ^Δflox^), as well as wild type alleles (*Pkd1* ^+^) in *Pkd1^Col1a1(3.6)-^* ^cKO^ (Fig. 1C). We found that *Col1a1(3.6)*-Cre-mediated floxed recombination occurred in both skeletal and nonskeletal tissues including pancreas, liver, and kidney (Fig. 1C), consistent with prior reports [31] that *Col1a1(3.6)*-Cre mRNA was highly expressed in calvarias, long bone, and tendon, but was also detected in brain, kidney, liver and lung [31].

To quantify the excised efficiency of floxed *Pkd1* by *Col1a1(3.6)*-Cre-recombinase, we examined the percentage of *Pkd1* transcripts in exons 2–4 in calvarias, kidney, and liver from 6-week-old mice by real-time RT-PCR. We found that *Col1a1(3.6)*-Cre;*Pkd1* ^flox/+^ mice exhibited approximately 18∼41% excision of the floxed exons 2–4 from total *Pkd1* transcripts, whereas *Pkd1^Col1a1(3.6)-^* ^cKO^ mice resulted in a net reduction of *Pkd1* expression by 45∼60% in calvarias, kidney, and liver (Fig. 1D, 1E, and 1F). In addition, there is a gene-dose-dependent reduction in *Pkd1* transcripts in calvaria (Fig. 1D) that correlated with a more severe bone phenotype in *Col1a1(3.6)*-Cre;*Pkd1* ^flox/null^ newborn mice compared to *Col1a1(3.6)*-Cre;*Pkd1* ^flox/flox^ mice.

### A gene dose-dependent effect of *Col1a1 (3.6)*-Cre-mediated conditional deletion of *Pkd1* in newborn and postnatal bone formation

**Figure 2:**
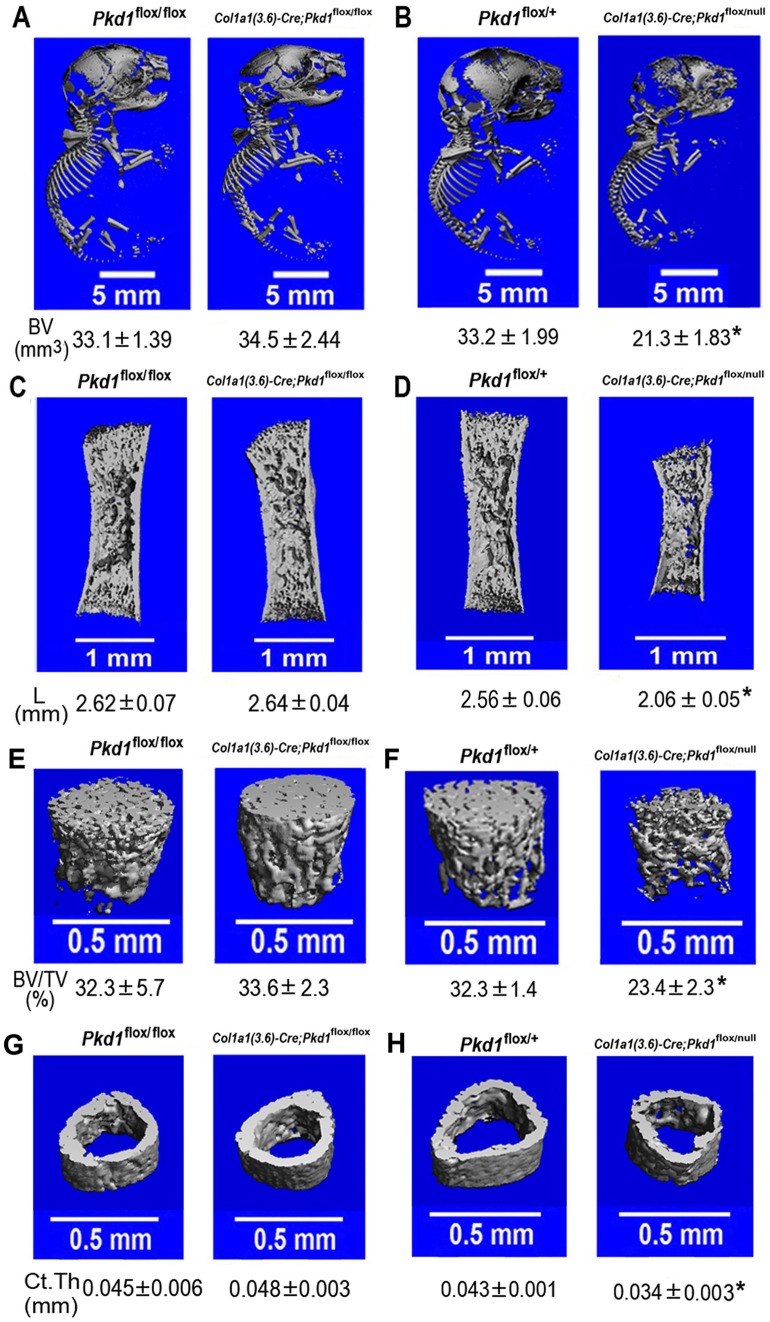
*Col1a1(3.6)*-Cre-mediated deletion of *Pkd1* results in osteopenia in *Col1a1(3.6)*-Cre;*Pkd1* ^flox/null^ newborn mice. The whole skeleton mineralization (A and B), full-length mineralized femurs (C and D), bone volume of metaphyseal region (E and F), and cortical thickness of cortical bone (G and H) of femurs from *Col1a1(3.6)*-Cre;*Pkd1* ^flox/flox^ and *Col1a(3.6)*-Cre; *Pkd1* ^flox/null^ newborn mice by μCT analysis. A *Pkd1* gene dose effect was observed during skeletogenesis between *Col1a1(3.6)*-Cre;*Pkd1* ^flox/flox^ and *Col1a(3.6)*-Cre; *Pkd1* ^flox/null^ newborn mice. Data represent the mean ± SD from three to four individual samples. *Significant difference from control mice (*Pkd1* ^flox/flox^ or *Pkd1* ^flox/+^) at *P<*0.05.

Indeed, we failed to observe abnormalities of skeletal development in homozygous *Pkd1^Col1a1(3.6)-^* ^cKO^ newborn mice (Fig. 2A, 2C, 2E and 2G). In contrast, bone structural abnormalities were observed in homozygous *Col1a1(3.6)*-Cre;*Pkd1* ^flox/null^ newborn mice, including delayed bone mineralization in calvarial and vertebral bone tissues (Fig. 2B), a short and less mineralized femur (Fig. 2D), and a significant reduction in both trabecular bone volume (Fig. 2F) and cortical bone thickness (Fig. 2H). Because the Cre;*Pkd1* ^flox/null^ strategy leads to greater Cre-mediated reduction in *Pkd1* conditional deletion compared to Cre;*Pkd1* ^flox/flox^ approach [33], the more severe phenotype that we observed is likely the result of greater reductions in *Pkd1* gene dose during embryogenesis.

**Figure 3:**
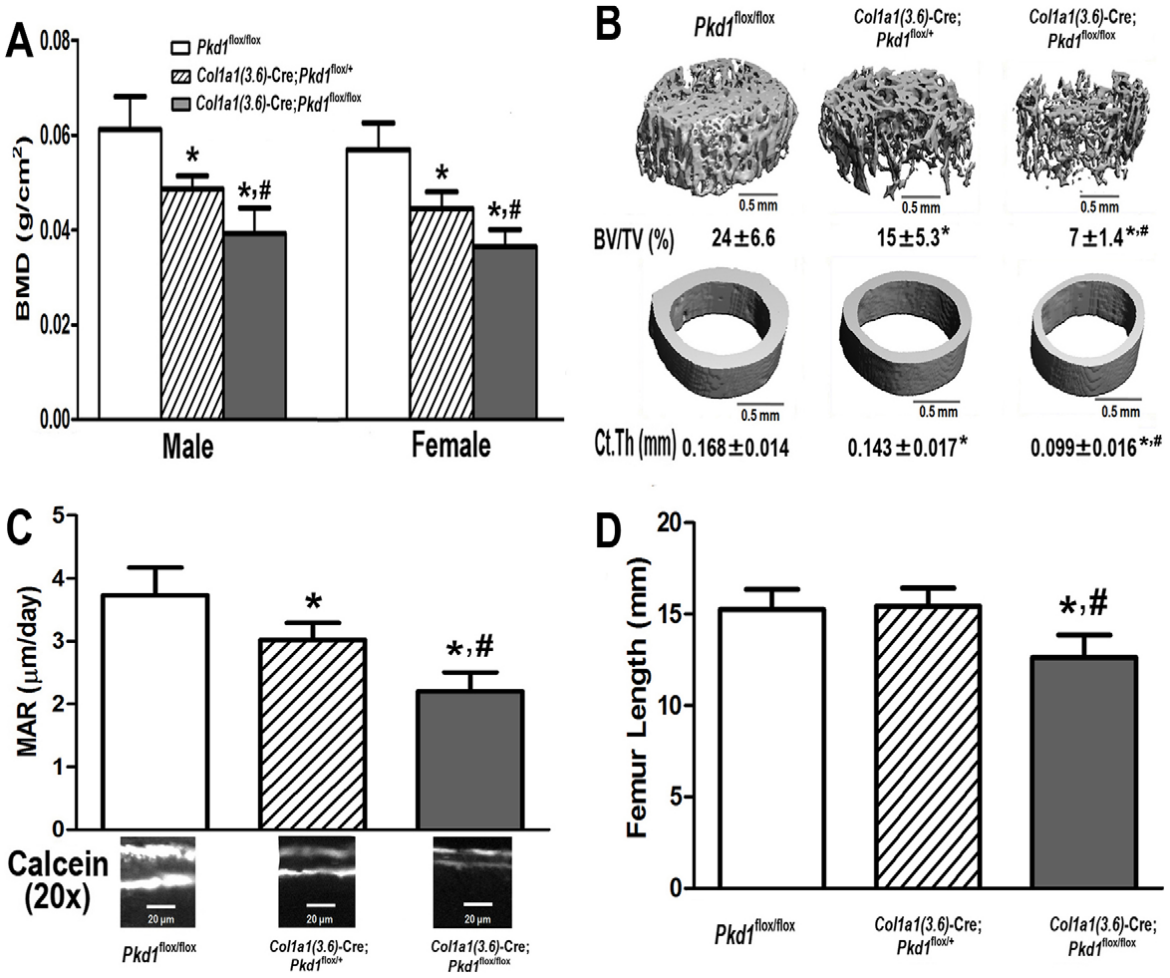
*Col1a1(3.6)*-Cre-mediated conditional deletion of *Pkd1* leads to severe osteopenia in *Col1a1(3.6)*-Cre;*Pkd1* ^flox/flox^ adult mice. (A) Bone mineral density (BMD), (B) Bone structure of femurs, (C) Bone mineral apposition rate (MAR), and (D) Femur length at 6 weeks of age. There was a *Pkd1* gene dose-dependent reduction in BMD in both male and female heterozygous *Col1a1(3.6)*-Cre; *Pkd1* ^flox/+^ and homozygous *Col1a1(3.6)*-Cre; *Pkd1* ^flox/flox^ mice compared with age-matched control mice (*Pkd1* ^flox/flox^). µCT analysis revealed that the lower bone mass in male *Col1a1(3.6)*-Cre-mediated mice with conditional deletion of *Pkd1* resulted from reductions in both trabecular BV/TV and cortical CtTh that were proportionate to the reduction of *Pkd1* gene dose. These reductions in bone mass and structure were associated with a 19% and 41% reduction in mineral apposition rate (MAR) in male heterozygous *Col1a1(3.6)*-Cre; *Pkd1* ^flox/+^ and homozygous *Col1a1(3.6)*-Cre; *Pkd1* ^flox/flox^ mice compared with age-matched control mice, respectively. In addition, the femurs of homozygous *Col1a1(3.6)*-Cre; *Pkd1* ^flox/flox^ mice were 17% shorter in length, indicating a postnatal bone growth retardation. Data represent the mean ± S.D. from five to six individual mice. *Significant difference from control (*Pkd1* ^flox/flox^) and ^#^significant difference from *Col1a1(3.6)*-Cre; *Pkd1* ^flox/+^ mice at *P<*0.05, respectively.

We also observed a *Pkd1* gene dose dependent reduction in bone mineral density (BMD) in heterozygous *Col1a1(3.6)*-Cre; *Pkd1* ^flox/+^ and homozygous *Pkd1^Col1a1(3.6)-^* ^cKO^ mice. A significant reduction in BMD of 21∼22% was observed in both male and female heterozygous *Col1a1(3.6)*-Cre;*Pkd1* ^flox/+^ mice at 6 weeks of age compared with age-matched control mice (*Pkd1* ^flox/flox^) (Fig. 3A). Homozygous *Pkd1^Col1a1(3.6)-^* ^cKO^ mice had greater loss in BMD, with respective reductions in BMD of 35% and 36% reduction in male and female adult mice (Fig. 3A). μCT analysis revealed that the lower bone mass in male heterozygous *Col1a1(3.6)*-Cre; *Pkd1* ^flox/+^ mice was caused by reduced trabecular bone volume (BV/TV, 40%) and cortical bone thickness (Ct.Th, 15%) (Fig. 3B), and homozygous *Col1a1(3.6)*-Cre;*Pkd1* ^flox/flox^ mice had greater loss in both trabecular (73%) and cortical bone (41%) than did heterozygous *Col1a1(3.6)*-Cre; *Pkd1* ^flox/+^ mice (Fig. 3B). These reductions in bone volume and cortical thickness were associated with a significant *Pkd1* gene dose-dependent decrease in periosteal mineral apposition rate (MAR)(Fig. 3C). In this regard, periosteal MAR was reduced by 19% in heterozygous *Col1a1(3.6)*-Cre; *Pkd1* ^flox/+^ mice and 41% in homozygous *Col1a1(3.6)*-Cre; *Pkd1* ^flox/flox^ mice compared with age-matched *Pkd1* ^flox/flox^ controls (Fig. 3C). In addition, the femurs of homozygous *Pkd1^Col1a1(3.6)-^* ^cKO^ mice were 17% shorter in length, indicating a role of Pkd1 in growth plate of metaphyseal bone (Fig. 3D). Interestingly, the severity of BMD reductions in more limited number of *Col1a1(3.6)*-Cre;*Pkd1* ^flox/null^ mice that were available for examination was similar to that of *Pkd1^Col1a1(3.6)-^* ^cKO^ mice (data not shown).

To investigate the effects of *Pkd1* deficiency on gene expression profiles in bone, we examined by real-time RT-PCR the expression levels of a panel of osteoblast lineage-, osteoclast-, and adipocyte-related mRNAs from the tibias of 6-week-old control *Pkd1* ^flox/flox^, heterozygous *Col1a1(3.6)*-Cre; *Pkd1* ^flox/+^, and homozygous *Pkd1^Col1a1(3.6)-^* ^cKO^ mice (Table 1). Consistent with a low bone mass phenotype by BMD and µCT analysis, we found a significant *Pkd1* gene dose-dependent decrease in osteoblast-lineage gene transcripts in these *Pkd1*-deficient mice, including *Runx2*, *Osterix, FGF23, Osteoprotegerin (Opg), Rank ligand*, and *alkaline phosphatase 2* (*Akp2*) mRNA levels, but no obvious change was observed in *osteocalcin* expression compared to control mice (Table 1). Consistent with a ratio of Opg/*RankL* , which would predict reduced osteoclastogenesis, bone expression of *tartrate-resistant acid phosphatase* (*Trap*) and matrix metallopeptidase 9 (*Mmp9*), markers of bone resorption, were also reduced in heterozygous *Col1a1(3.6)*-Cre; *Pkd1* ^flox/+^ and homozygous *Pkd1^Col1a1(3.6)-^* ^cKO^ mice (Table 1). The changes in bone mRNA expression did not correlate with serum levels of these biomarkers, except serum RankL, which was reduced in *Pkd1^Col1a1(3.6)^* ^-cKO^ mice. Serum osteocalcin and OPG were elevated and TRAP was in the normal range in homozygous *Pkd1^Col1a1(3.6)^* ^-cKO^ mice compared to age-matched control *Pkd1* ^flox/flox^ (Table 2).

**Table 1:** Gene-expression profiles in 6-week-old mice.

Gene	Accession no.	*Pkd1* ^flox/flox^	*Col1a1(3.6)-Cre; Pkd1* ^flox/+^	*Col1a1(3.6)-Cre; Pkd1* ^flox/flox^	*p*-value
**Osteoblast lineage**					
*Pkd1*	NM_013630	1.00±0.29	0.75±0.21*	0.53±0.19*^,#^	0.0005
*Runx*2	NM_009820	1.00±0.25	0.76±0.17*	0.54±0.11*^,#^	0.0003
*Osterix*	NM_130458	1.00±0.24	0.78±0.21*	0.49±0.11*^,#^	0.0005
*Osteocalcin*	NM_007541	1.00±0.43	0.90±0.33	1.10±0.76	0.8078
*Opg*	MMU94331	1.00±0.25	0.94±0.37	0.45±0.24*^,#^	0.0041
*Rank ligand*	NM_011613	1.00±0.34	0.58±0.19*	0.26±0.13*^,#^	0.0002
*Akp2*	NM_007431	1.00±0.27	0.74±0.21*	0.48±0.11*^,#^	0.0006
*FGF23*	NM_022657	1.00±0.33	0.84±0.11	0.56±0.21*^,#^	0.0076
**Osteoclast**					
*Trap*	NM_007388	1.00±0.31	0.73±0.15*	0.40±0.13*^,#^	0.0007
*Mmp*9	NM_013599	1.00±0.41	0.68±0.12*	0.44±0.11*^,#^	0.0006
**Adipocyte**					
*Adiponectin*	NM_009505	1.00±0.21	1.04±0.43	1.82±0.56*^,#^	0.0077
*aP2*	NM_024406	1.00±0.36	1.22±0.33	2.45±1.51*^,#^	0.0268
*Lpl*	NM_008509	1.00±0.15	1.17±0.21	1.63±0.36*^,#^	0.0010

Data are mean ±S.D. from 5–6 tibias of 6-week-old individual mice and expressed as the fold changes relative to the housekeeping gene *β-actin* subsequently normalized to control mice. * indicates significant difference from control *Pkd1* ^flox/flox^ mice, and ^#^ indicates significant difference from heterozygous *Col1a1(3.6)*-Cre;*Pkd1* ^flox/+^ mice at *p<*0.05, respectively.

**Table 2:** Biochemistry analysis of serum in 6-week-old mice.

Genotype	*Pkd1* ^flox/flox^	*Col1a1(3.6)-Cre;Pkd1* ^flox/+^	*Col1a1(3.6)-Cre;Pkd1* ^flox/flox^
BUN(mg/dl)	21±1.8	20±2.8	36±10.4*^,#^
Ca (mg/dl)	9.6±0.24	9.9±0.31	9.9±0.59
P (mg/dl)	7.3±0.42	7.1±0.15	6.6±0.24*,#
Osteocalcin (ηg/ml)	40±17	88±26	169±91*,#
OPG (pg/ml)	3.2±0.38	3.6±0.51	5.1±1.15*,#
RankL (pg/ml)	89±24	94±19	53±23*,#
TRAP (U/L)	4.0±0.43	3.5±0.82	3.6±0.83
PTH (pg/ml)	42±16	40±5.6	92±54*,#
FGF23 (pg.ml)	94±25	88±24	173±99*,#

Data are mean ± S.D. from 6–8 individual mice. * and # indicates significant difference from control *Pkd1* ^flox/flox^ and *Col1a1(3.6)*-Cre;*Pkd1* ^flox/+^ mice at *p<*0.05, respectively. Osteocalcin is produced by osteoblasts, and TRAP is produced by osteoclasts.

**Figure 4:**
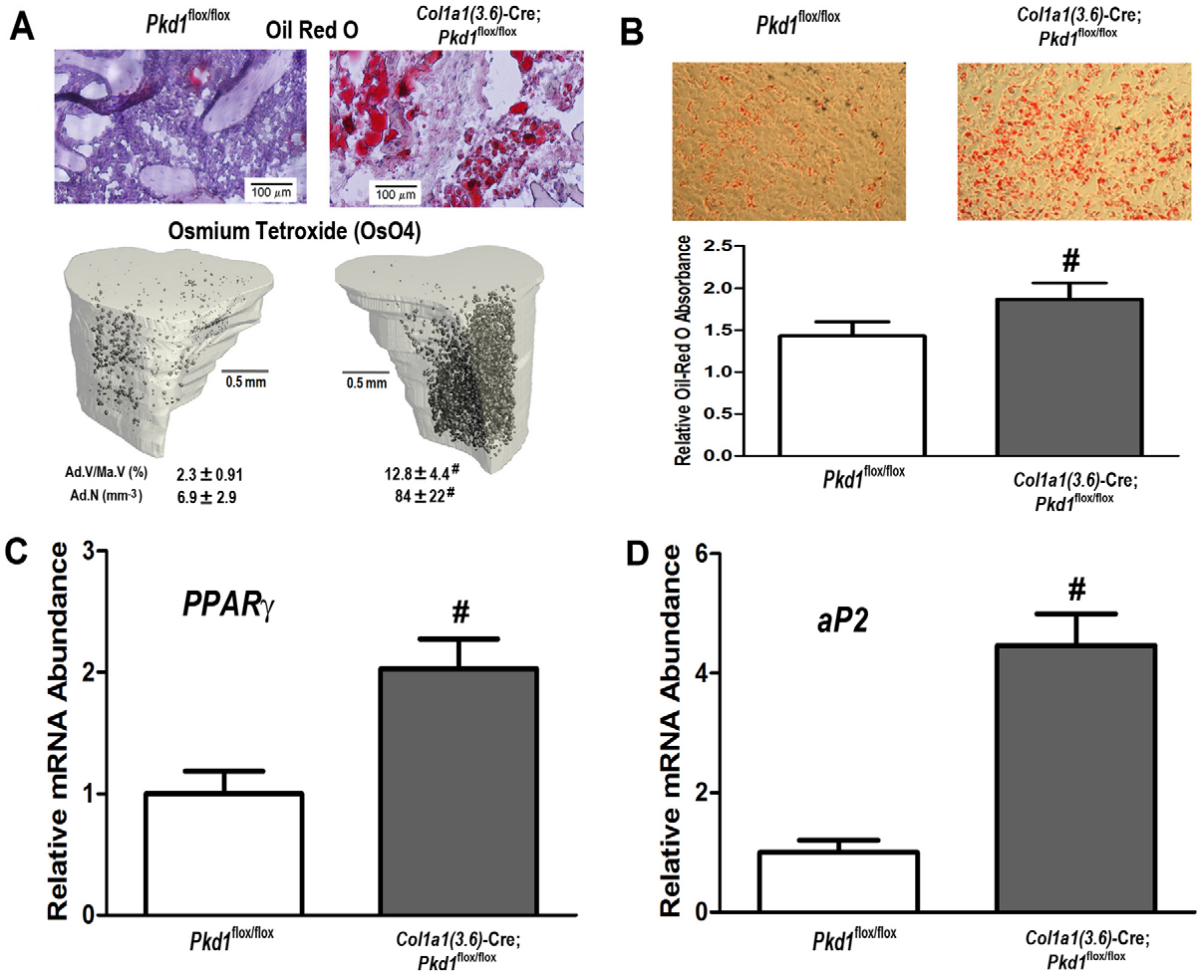
*Col1a1(3.6)*-Cre-mediated conditional deletion of *Pkd1* results in enhanced adipogenesis in bone marrow and in bone stromal cell cultures. (A) Histology of adipocytes in decalcified tibias. Oil Red O staining (upper panel) showed that the numbers of adipocytes and fat droplets in tibia bone marrow were greater in 6-week-old *Col1a1(3.6)*-Cre; *Pkd1* ^flox/flox^ mice compared with age-matched control *Pkd1* ^flox/flox^ mice. Osmium tetroxide (OsO4) staining by μCT analyses (lower panel) showed that adipocyte volume/marrow volume (Ad.V/Ma.V, %) and adipocyte number (Ad.N, mm^−3^) were much higher in the proximal tibia from 6-week-old *Col1a1(3.6)*-Cre; *Pkd1* ^flox/flox^ mice compared with age-matched control *Pkd1* ^flox/flox^ mice. (B) Adipocytic differentiation in BMSC cultures. An increase of adipogenesis potential was observed in 6-week-old *Col1a1(3.6)*-Cre; *Pkd1* ^flox/flox^ BMSC cultures, evidenced by a significant increase of Oil Red O staining in adipogenic cultures. (C and D) Expression of adipogenic markers by real-time RT-PCR. Significantly increased levels of *PPARγ* and *aP2* mRNAs were observed in 6-week-old *Col1a1(3.6)*-Cre; *Pkd1* ^flox/flox^ BMSC cultures compared with control (*Pkd1* ^flox/flox^) cultures. Data are expressed as the mean ± SD from three independent experiments. ^#^ Significant difference from control (*Pkd1* ^flox/flox^) at *P<*0.05.

In accordance with decreased osteogenesis in bone, we also observed an increased adipogenesis in bone marrow and in bone marrow stromal cultures from homozygous *Pkd1^Col1a1(3.6)-^* ^cKO^ mice. In this regard, homozygous *Pkd1^Col1a1(3.6)-^* ^cKO^ mice showed a higher number of adipocytes and volume of fat droplets in decalcified tibias stained with Oil Red O and OsO4 (Fig. 4A). BMSC cultures derived from *Pkd1^Col1a1(3.6)-^* ^cKO^ mice exhibited a marked increase of Oil Red O stained adipocytes (Fig. 4B). In addition, *PPAR*γ, an adipocyte transcription factor, and adipocyte markers such as *aP*2 (adipocyte fatty acid-binding protein 2) were also significantly increased in BMSC cultures of *Pkd1^Col1a1(3.6)-^* ^cKO^ mice compared to *Pkd1* ^flox/flox^ control mice (Fig. 4C and 4D), consistent with increased adipogenic markers including *adiponectin*, *aP2*, and *Lpl* (lipoprotein lipase) in long bone samples of *Pkd1^Col1a1(3.6)-^* ^cKO^ mice (Table 1).

### Effect of *Col1a1 (3.6)*-Cre-mediated conditional deletion of *Pkd1* on osteoblastic function *ex vivo*

**Figure 5:**
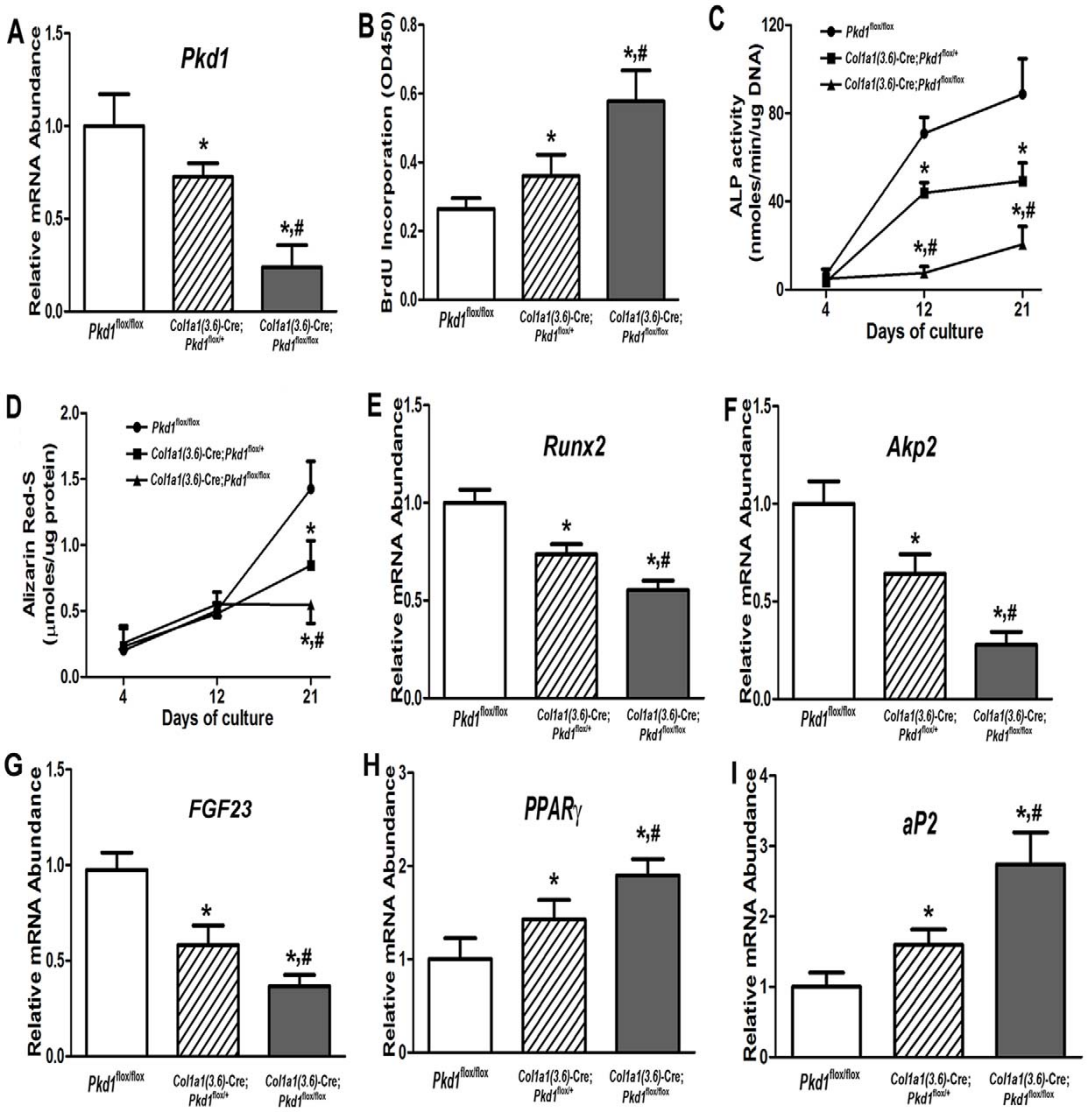
Effects of *Col1a1(3.6)*-Cre-mediated conditional deletion of *Pkd1* on osteoblastic proliferation and maturation, as well as gene expression profiles *ex vivo*. (A) Total *Pkd1* transcripts by real-time RT-PCR. All *Pkd1* transcripts were dose-dependently reduced in primary osteoblast cultures from heterozygous *Col1a1(3.6)*-Cre; *Pkd1* ^flox/+^ and homozygous *Col1a1(3.6)*-Cre; *Pkd1* ^flox/flox^ mice. (B) BrdU incorporation. A gene dose-dependent increase of BrdU incorporation was observed during 6 h of primary osteoblast culture from heterozygous *Col1a1(3.6)*-Cre; *Pkd1* ^flox/+^ and homozygous *Col1a1(3.6)*-Cre; *Pkd1* ^flox/flox^ mice. (C) ALP activity. Osteoblasts from control, heterozygous, and homozygous mice displayed time-dependent increments in ALP activity during 21 days of culture, but ALP activity was gene dose- and time-dependently decreased in heterozygous and homozygous osteoblasts compared to control osteoblasts. (D) Quantification of mineralization. Alizarin Red-S was extracted with 10% cetylpyridinium chloride and quantified as described in Materials and Methods. A time-dependent increment of Alizarin Red-S accumulation was observed in control, heterozygous, and homozygous osteoblasts during 21 days of culture, but the accumulation was gene dose-dependently decreased in heterozygous and homozygous osteoblasts cultures compared to control osteoblasts at day 21 of culture. (*E–I*) Gene expression profiles by real-time RT-PCR. Osteoblastic markers such as *Runx2*, *Akp2*, and *FGF23* were gene dose-dependently reduced during 18 days of osteogenic culture from heterozygous and homozygous osteoblasts. In contrast, a marked increase of adipocyte markers such as *PPARγ2* and *aP2* was observed from heterozygous and homozygous osteoblasts under the same osteogenic media when compared with control osteoblasts. Data are expressed as the mean ± SD from three independent experiments. *Significant difference from control (*Pkd1* ^flox/flox^); ^#^significant difference from heterozygous *Col1a1(3.6)*-Cre; *Pkd1* ^flox/+^ mice at *P<*0.05, respectively.

To determine the impact of *Col1a1(3.6)*-Cre-mediated conditional deletion of *Pkd1* on osteoblast function *ex vivo*, we isolated primary calvarial osteoblasts from E17.5 control *Pkd1* ^flox/flox^, heterozygous *Col1a1(3.6)*-Cre; *Pkd1* ^flox/+^ , and homozygous *Pkd1^Col1a1(3.6)-^* ^cKO^ fetuses. Primary calvarial osteoblasts under osteogenic culture condition undergo progressive alterations in cell proliferation and osteoblastic differentiation that recapitulates the osteoblastic developmental program [34]. Consistent with our previous report [17], we found that heterozygous *Col1a1(3.6)*-Cre;*Pkd1* ^flox/+^ and homozygous *Pkd1^Col1a1(3.6)^* ^-cKO^ osteoblasts increased BrdU incorporation that were proportionate to the reduction of *Pkd1* gene dose (Fig. 5A and 5B). In addition, heterozygous *Col1a1(3.6)*-Cre;*Pkd1* ^flox/+^ and homozygous *Pkd1^Col1a1(3.6)-^* ^cKO^ osteoblasts had a gene dose-dependent impairment of osteoblastic differentiation and maturation, as evidenced by culture duration-dependent reductions in ALP activity (Fig. 5C), diminished calcium deposition in extracellular matrix (Fig. 5D), and reduced osteoblastic differentiation markers, including *Runx2* and *Akp2*, compared to control *Pkd1* ^flox/flox^ osteoblasts (Fig. 5E and 5F). A similar reduction of *FGF23* transcripts was also observed *in vitro* cultures osteoblasts at day 18 (Fig. 5 G). In agreement with increased adipogenic markers observed in bone *in vivo*, heterozygous *Col1a1(3.6)*-Cre;*Pkd1* ^flox/+^ and homozygous *Pkd1^Col1a1(3.6)-^* ^cKO^ osteoblasts exhibited a gene dose-dependent increase of adipocyte markers such as *PPARγ2* and *aP2* (Fig. 5H and 5I), suggesting that impairment of osteogenesis was associated with enhancement of adipogenesis in *Col1a1(3.6)*-Cre-mediated conditional deletion of *Pkd1* osteoblast cultures.

### Development of polycystic kidney and pancreatic disease in homozygous *Pkd1^Col1a1(3.6)-^* ^cKO^ embryos and mice

**Figure 6:**
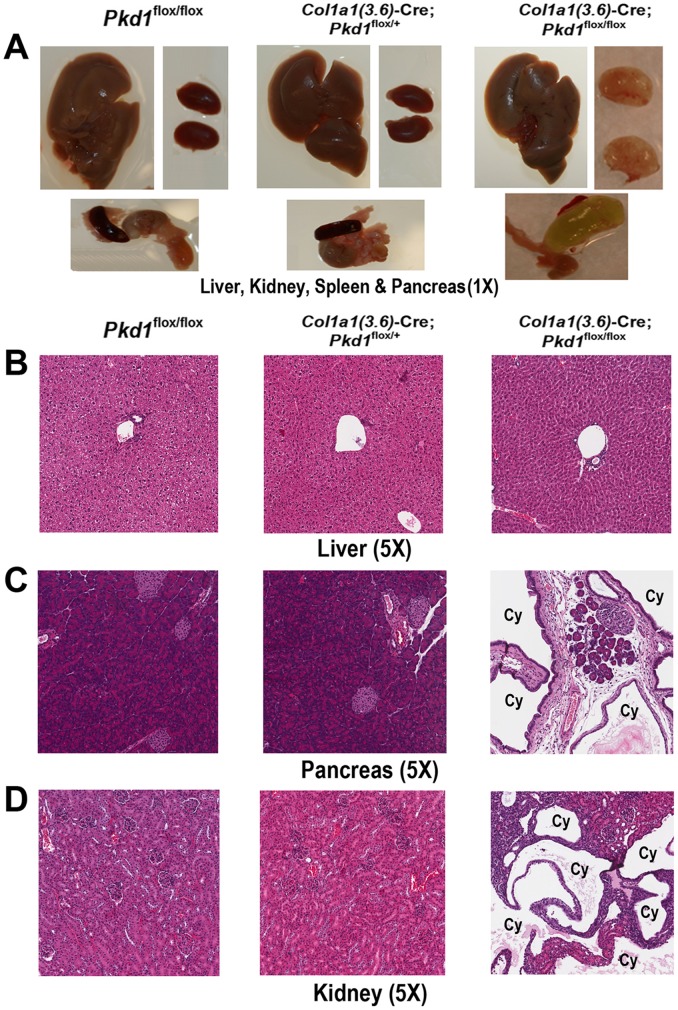
*Col1a1(3.6)*-Cre-mediated conditional deletion of *Pkd1* causes polycystic pancreas and kidney. (A) Gross appearance of liver, kidney, and pancreas. There was no cyst formation in the liver, kidney, and pancreas in heterozygous *Col1a1(3.6)*-Cre;*Pkd1* ^flox/+^ mice, whereas age-matched homozygous *Col1a1(3.6)*-Cre;*Pkd1* ^flox/flox^ mice developed severe renal and pancreatic cysts at 6 weeks of age. (B–D) Hematoxylin-eosin-stained sections (5X) of liver, pancreas, and kidney from 6-week-old mice. Cysts were not observed in the livers from heterozygous and homozygous mice, and renal and pancreatic cysts were also not found in kidney and pancreas tissues from heterozygous *Col1a1(3.6)*-Cre;*Pkd1* ^flox/+^ mice. However, homozygous *Col1a1(3.6)*-Cre;*Pkd1* ^flox/flox^ mice exhibited massive cyst formation in both the pancreas and kidney. Interestingly, glomeruli formation in the kidney and endocrine islet formation in pancreas appeared to be unaffected. In addition, expansion of pancreatic ducts formed large pancreatic cysts that led to massive acinar cell loss, formation of abnormal tubular structures, and appearance of endocrine cells in ducts.

Besides skeletal abnormalities, we also observed that homozygous *Pkd1^Col1a1(3.6)-^* ^cKO^ mice developed severe renal and pancreatic cysts at 6 weeks-of-age. In contrast, no cyst formation was observed in the kidney or pancreas of age-matched control *Pkd1* ^flox/flox^ or heterozygous *Col1a1(3.6)*-Cre;*Pkd1* ^flox/+^ mice (Fig. 6A). Hematoxylin-eosin (H&E)-stained sections showed no cyst formation in liver tissues from all of three groups (Fig. 6B), in spite of 35% and 54% reduction in *Pkd1* mRNA expression in heterozygous *Col1a1(3.6)*-Cre;*Pkd1* ^flox/+^ and homozygous *Col1a1(3.6)*-Cre;*Pkd1* ^flox/flox^ mice, respectively (Fig. 1F). Homozygous *Pkd1^Col1a1(3.6)-^* ^cKO^ mice developed massive cysts in both the pancreas and kidney; however, glomeruli formation in the kidney and endocrine islet formation in pancreas were unaffected (Fig. 6C and 6D). Expansion of pancreatic ducts formed large pancreatic cysts that led to massive acinar cell loss, formation of abnormal tubular structures, and appearance of endocrine cells in ducts (Fig. 6C and 6D).

**Figure 7:**
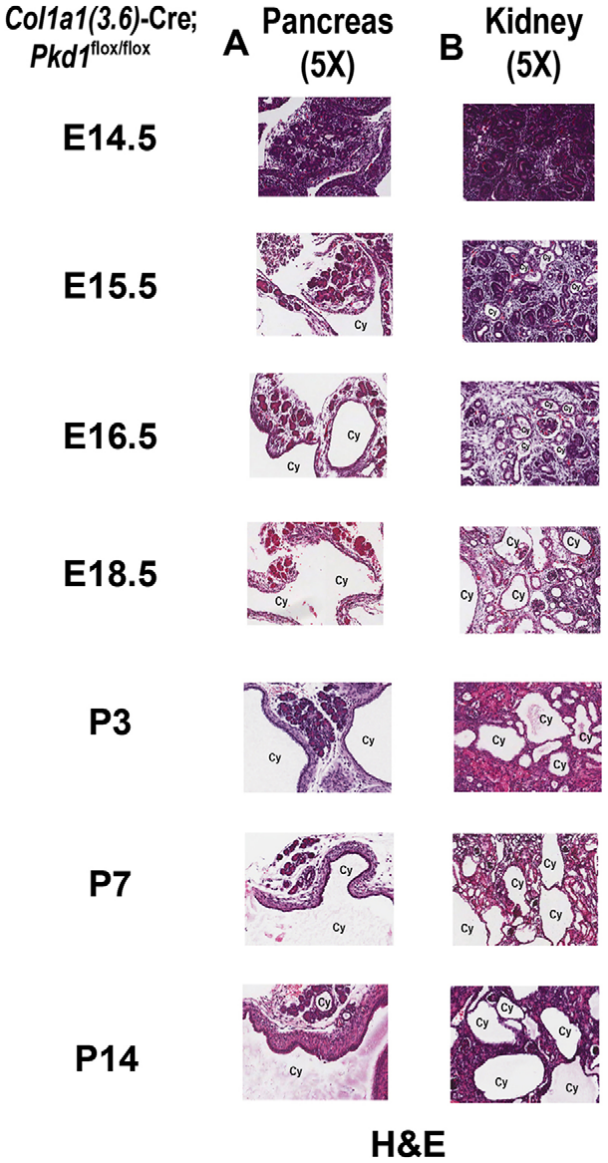
Development of cysts in pancreas and kidney caused by *Col1a1(3.6)*-Cre-mediated conditional deletion of *Pkd1*. Hematoxylin-eosin (H&E) staining (5X) for pancreas (A) and kidney (B) between E14.5 and P14. Both pancreatic duct and renal tubule cysts started at E15.5 in homozygous *Col1a1(3.6)*-Cre;*Pkd1* ^flox/flox^ embryos, and the size of renal cystic lesions developed rapidly between E15.5 and P14.

**Figure 8:**
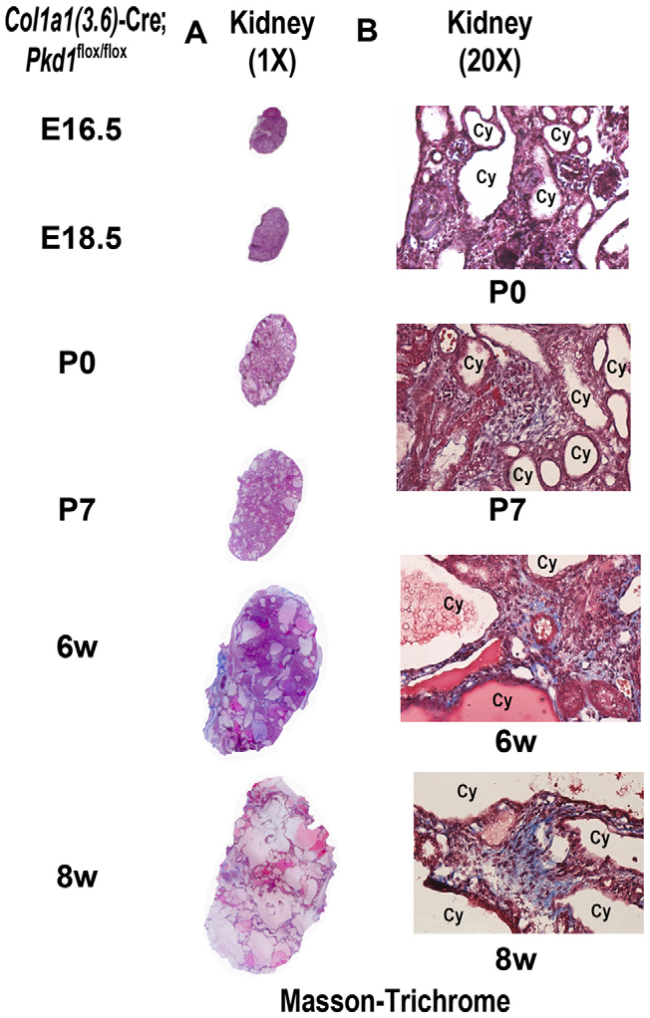
Development of fibrosis in kidney caused by *Col1a1(3.6)*-Cre-mediated conditional deletion of *Pkd1*. Masson-trichrome staining for fibrosis in polycystic kidney sections (A)1X, (B) 20X magnification between E16.5 and 8 weeks (8w). Masson-trichrome staining was observed in polycystic kidney tissue at P7 and became more severe at 8 weeks of age, indicating a renal fibrosis formation occurring in *Col1a1(3.6)*-Cre;*Pkd1* ^flox/flox^ mice.

**Figure 9:**
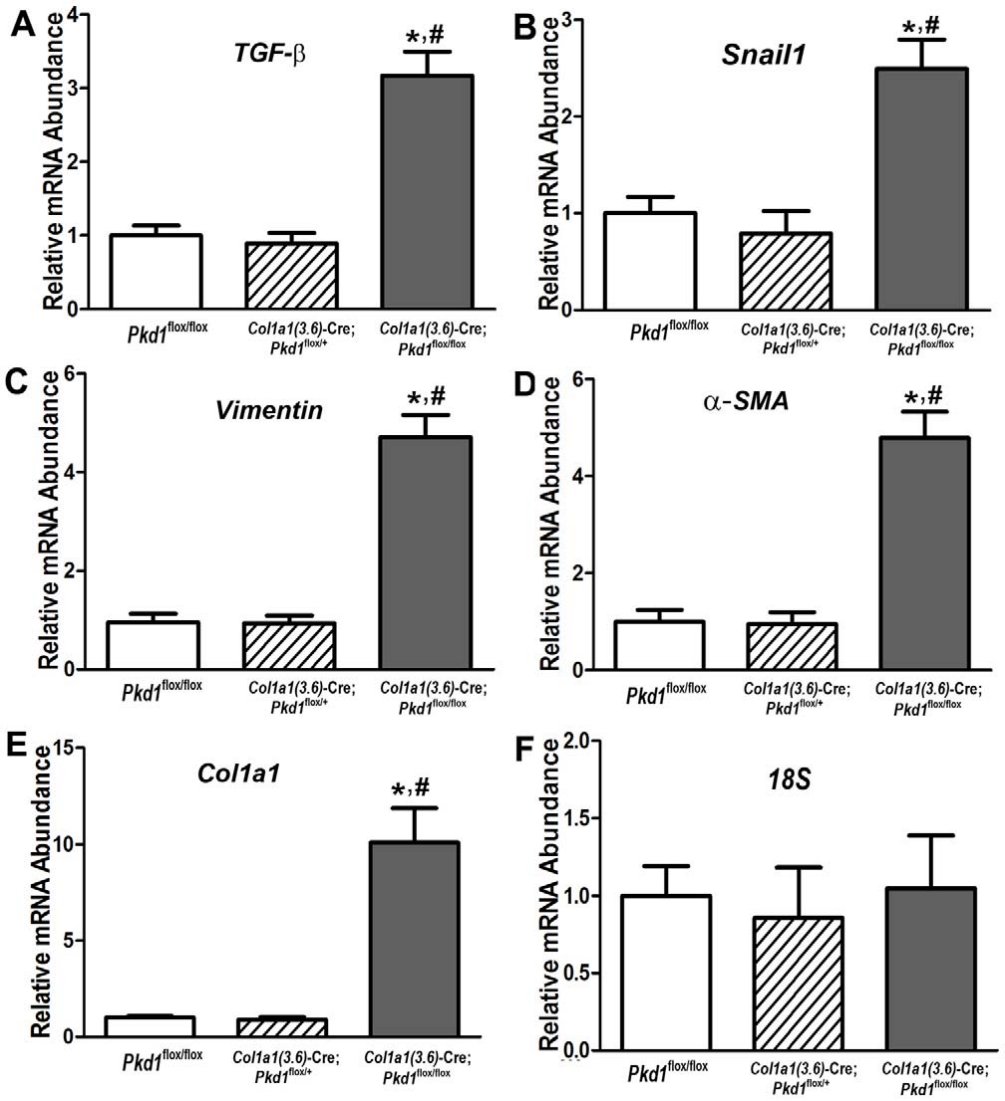
Effect of *Col1a1(3.6)*-Cre-mediated conditional deletion of *Pkd1* in kidney on expression of epithelia-mesenchymal-transition (EMT) and fibrosis markers. (A–C) Expression of EMT markers in polycystic kidneys of 6-week-old mice by real-time RT-PCR. A panel of EMT markers including transforming growth factor β (TGF-β), *snail1*, and *vimentin* were significantly up-regulated in polycystic kidney from homozygous *Col1a1(3.6)*-Cre;*Pkd1* ^flox/flox^ mice compare with age-matched control *Pkd1* ^flox/flox^ mice. (D–F) Expression of fibrosis markers in polycystic kidneys of 6-week-old mice by real-time RT-PCR. A panel of fibrosis markers such as α-smooth muscle actin (α-SMA) and precollagen type I (*Col1a1*) was markedly increased in the kidney from homozygous *Col1a1(3.6)*-Cre;*Pkd1* ^flox/flox^ mice compare with age-matched control *Pkd1* ^flox/flox^ mice. 18 S was served as an internal control for gene expressions. There were no differences in EMT and fibrosis markers between heterozygous and control *Pkd1* ^flox/flox^ mice. Data represent the mean ± SD from five to six individual mice. *Significant difference from control (*Pkd1* ^flox/flox^); ^#^significant difference from *Col1a1(3.6)*-Cre;*Pkd1* ^flox/+^ mice at *P<*0.05, respectively.

Timed pregnancies were generated to analyze heterozygous *Col1a1(3.6)*-Cre;*Pkd1* ^flox/+^ and homozygous *Pkd1^Col1a1(3.6)-^* ^cKO^ fetuses at various developmental stages. We did not observe renal or pancreatic cysts in heterozygous *Col1a1(3.6)*-Cre;*Pkd1* ^flox/+^ mice during embryogenesis or at 6-weeks of age (data not shown). Pancreatic cysts first became evident in homozygous *Pkd1^Col1a1(3.6)-^* ^cKO^ embryos was at E15.5 (Fig. 7A and 7B). In addition, the dilatation of pancreatic cysts progressed as a function of age in homozygous *Pkd1^Col1a1(3.6)^* ^-cKO^ (Fig. 7A and 7B). The size of renal cystic lesions also increased as a function of age. Masson-trichrome stained sections of the kidney showed that the cystic fibrosis of polycystic kidney started at P7 and became progressively more severe by 6-weeks of age (Fig. 8A and 8B). Real-time RT-PCR showed that mesenchymal-to-epithelial transition and fibrosis formation genes were significantly up-regulated in cystic kidney from homozygous *Pkd1^Col1a1(3.6)^* ^-cKO^ mice compare with control *Pkd1* ^flox/flox^ mice (Fig. 9A–9F), but no such alterations were observed in heterozygous *Col1a1(3.6)*-Cre;*Pkd1* ^flox/+^ mice (Fig. 9A–9F). In addition, homozygous *Pkd1^Col1a1(3.6)-^* ^cKO^ mice had significantly higher levels of serum BUN, PTH, and FGF23, but lower levels of phosphorus, and no changes in calcium levels at 6 weeks of age (Table 2), consistent with the development of renal impairment caused by polycystic kidney disease and secondary induction of compensatory hormonal changes (Table 2).

## Discussion

Pkd1, Pkd2, and primary cilia are present in mature osteoblasts and osteocytes [16], where the primary cilia-polycystin complex plays an important role in postnatal osteoblast and osteocyte regulation of bone formation and mechanosensing [17], [18]. Primary cilia-polycystin complexes are known to have a role in regulating developmental pathways in other tissues, such as left-right patterning in embryos and kidney development [35], but the role of Pkd1 in osteoblast development and bone embryogenesis, while suggested by skeletal alterations in global *Pkd1* knockout mice [16], [17], [18], [36], has not been confirmed by selective ablation of *Pkd1* early in the osteoblast lineage. In the current study, *Pkd1* was conditionally deleted in mesenchymal precursors that are destined to become multiple cell types including osteoblast lineage [37], renal tubule cells [38], pancreas duct epithelial cells [39], and bile ductal plate cells [40] by creating *Pkd1^Col1a1(3.6)-^* ^cKO^ or *Col1a1(3.6)*-Cre;*Pkd1* ^flox/null^ mice, which differ in the degree of *Pkd1* deletion [33].

We found that reduction of *Pkd1* in mesenchymal precursors in *Pkd1^Col1a1(3.6)-^* ^cKO^ mice resulted in impaired osteoblast-mediated bone formation and low bone mass in the adult mouse, consistent with the observations in *Oc*-Cre- and *Dmp1*-Cre-mediated conditional knockout of *Pkd1* [17], [18]. Primary osteoblast cultures showed higher cell replication and lower osteoblastic differentiation markers in E17.5 homozygous *Pkd1^Col1a1(3.6)-^* ^cKO^ mice, similar to the defects of osteoblastic maturation in *Oc*-Cre-mediated conditional knockout of *Pkd1* [17]. The increased proliferation observed in *Pkd1*-deficient osteoblasts is similar to increased renal cell proliferation caused by loss of *Pkd1* in renal epithelial cells, suggesting that an important function of Pkd1 is to regulate cell proliferation, which is typically inversely correlated with differentiation [41], [42]. In addition, the marrow fat content of bone was increased and enhanced adipogenesis was found in bone marrow stromal cell cultures, which expressed decreased levels of the osteoblast differentiation factor *Runx2* and increased levels of *PPARγ* (peroxisome-proliferator-activated receptor-γ), which regulates adipocyte development. These findings are consistent with previous studies showing that Pkd1 stimulates osteogenesis and inhibits adipogenesis through a Pkd2-calcium dependent *Runx2* expression and that compound heterozygous *Pkd1* and *Runx2* deficient mice have additive effects to induce osteopenia [17], [18], [36]. Further studies are needed to determine the signaling pathways linking *Pkd1* inactivation in pre-osteoblasts to increments in *PPARγ* expression.

We were surprised by the difference in skeletal abnormalities between newborn *Col1a1(3.6)*-Cre;*Pkd1* ^flox/null^ and *Pkd1^Col1a1(3.6)-^* ^cKO^ mice. *Col1a1(3.6)*-Cre;*Pkd1* ^flox/null^ had evidence of abnormal skeletogenesis, albeit less severe than the previously described global *Pkd1* ^−/−^ mouse models [14], [15], [16]. *Col1a1(3.6)*-Cre, which is highly active between E12.5 and E14.5, and the resulting bone abnormalities are consistent with known effects of Pkd1to regulate *Runx2*, an essential transcriptional factor controlling osteoblast development [43], [44]. In contrast, newborn *Pkd1^Col1a1(3.6)-^* ^cKO^ mice lacked a demonstrable bone phenotype, consistent with studies in *Osx*-Cre;*Pkd1* ^flox/flox^ mice, which also failed to find evidence for a role of *Pkd1* in skeletogenesis [45]. A gene-dose dependent effect on skeletogenesis and differences in methods of conditional gene targeting might explain these discrepancies. In this regard, the less severe skeletal phenotype in newborn *Pkd1^Col1a1(3.6)-^* ^cKO^ and *Osx*-Cre;*Pkd1* ^flox/flox^ mice compared to *Col1a(3.6)*-Cre;*Pkd1* ^flox/null^ mice may be due to insufficient reductions in *Pkd1* expression to cause abnormal osteoblast development [33]. Additional studies are needed using the heterozygous floxed allele paired with a “null” mutant allele along with earlier osteoblast lineage specific and less leaky promoters to define the function of Pkd1 in osteoblast development during different stages of embryogenesis [17], [33], [46].

The role of Pkd1 in post-natal bone remodeling is further documented by the current studies. Bone remodeling that occurs postnatally is characterized by the recruitment of bone marrow mesenchymal stem cells to differentiate into osteoblasts that refill resorptive cavities. Our studies suggest that a reduction of *Pkd1* expression of more than 50% results in abnormalities in osteoblast-mediated bone formation in adult mice through *Pkd1* regulation of critical transcription factors involved in osteoblastogenesis and adipogenesis [47], [48], [49]. We have previously shown that the bone-specific deletion of *Pkd1* using *Oc*-Cre or *Dmp1*-Cre had a direct role in adult bone formation [17], [18]. However, we observed a more severe osteopenia in adult *Pkd1^Col1a1(3.6)-^* ^cKO^ compared to *Oc*-Cre or *Dmp1*-Cre-mediated *Pkd1* deletion, which could result from the effects of *Col1a1(3.6)*-Cre to delete *Pkd1* during embryogenesis or to the leakiness of this promoter leading to alterations in systemic factors that lead to secondary effects on bone. Although we observed significant increases of serum PTH and FGF23 levels in association with elevated BUN in adult *Pkd1^Col1a1(3.6)-^* ^cKO^ mice, the expected PTH-induced increases in bone transcripts were not observed, rather we found that *Akp2*, *FGF23*, *Trap*, *Osterix* and *Runx2* transcripts were decreased in *Pkd1^Col1a1(3.6)-^* ^cKO^, suggesting a direct effect of Pkd1 on bone. In addition, our finding of abnormal skeletogenesis in newborn *Col1a1(3.6)*-Cre;*Pkd1* ^flox/null^ mice and impairment in osteoblast differentiation maturation in E17.5 immortalized osteoblast cultures from *Pkd1^Col1a1(3.6)-^* ^cKO^ mice, are consistent with direct effects of Pkd1 in osteoblasts, as previously reported [16].

Interestingly, in this model, serum phosphate was low and FGF23 was high, consistent with other reports that FGF23 regulation may be abnormal in ADPKD [19]. However, we failed to find evidence for increased *FGF23* mRNA expression in bone or in osteoblasts cultures derived from *Pkd1^Col1a1(3.6)-^* ^cKO^ mice; rather *FGF23* mRNA levels were decreased in bone and osteoblasts cultures from these mice, suggesting that reduction of *Pkd1* in osteoblast lineage diminishes FGF23 production. The disparity between serum FGF23 and bone expression of *FGF23* is consistent with recent findings that FGF23 is regulated by both transcriptional and post-transcriptional mechanisms [50].

In addition to skeletal abnormalities, we found that *Col1a1(3.6)*-Cre-mediated deletion of *Pkd1* resulted in cyst formation in the kidney and pancreas. Although *Pkd1* is expressed in undifferentiated mesenchyme during embryogenesis [1], [2], [51] and Pkd1 mutations lead to cyst formation in the kidney, pancreas and liver in hereditary polycystic diseases [52], [53], [54], [55], [56], extraskeletal abnormalities due to deletion of *Pkd1* in mesenchymal precursors using *Col1a1(3.6)*-Cre was unexpected. Indeed, the *Col1a1(3.6)* promoter has been purported to specifically target cells of the osteoblastic lineage, and in previous reports was found to have minimal expression of Cre-recombinase in liver, pancreas, and kidney [30], [32]. However, unlike our studies, the prior reports did not examine Cre-mediated recombination in extraskeletal tissues. Indeed, we found evidence for *Col1a1(3.6)*-Cre deletion of *Pkd1* in all tissues tested, including kidney, pancreas and liver. Pancreatic and renal cysts developed by E15.5, just after highly expression of *Col1a1(3.6)*-Cre activity in skeletal tissues at E12.5 [29], [30], [31]. The size of polycystic pancreas and kidney varied between individual mice, and only 30% of individual homozygous *Pkd1^Col1a1(3.6)-^* ^cKO^ mice had both polycystic pancreas and kidney, indicating the possible effects of a mixed genetic background or other factors affecting cyst formation in these mice. This observation, along with the fact that mesenchymal cells give rise to both osteoblasts during bone development and renal tubular epithelia through mesenchymal-to-epithelial transition during kidney development [37], [57], [58], [59], and pancreatic mesenchyme gives rise to pancreatic ducts by epithelial-mesenchymal interaction during pancreas development [60], [61], [62], suggests a broad role of Pkd1 in mesenchymal development pathways. Also, consistent with the known gene-dose dependent effect of *Pkd1* in cystogenesis that requires a second hit in humans to cause ADPKD, there was no evidence of cysts formation in the pancreas or kidney of heterozygous *Col1a1(3.6)*-Cre;*Pkd1* ^flox/+^ mice. Approximately 30% of homozygous *Pkd1^Col1a1(3.6)-^* ^cKO^ adult mice exhibited a polycystic pancreas containing solitary unilocular cysts, whereas pancreatic cysts have been reported in only 5% of patients with ADPKD [63], [64].

Interestingly, we did not observe cyst formation in the liver of 6-week-old homozygous *Pkd1^Col1a1(3.6)-^* ^cKO^ mice, in spite of the known propensity for liver cyst formation in hereditary cystic disorders. The reason for the absence of cyst formation in the liver is not clear from our studies, because *Col1a1(3.6)*-Cre is expressed in the liver and results in excision of the *Pkd1* flox allele in this tissue, similar to the pancreas and kidney. In contrast, *MTTV*-Cre-mediated conditional deletion of *Pkd1* results in liver cysts by 10 weeks-of-age [25], suggesting that the lack of liver cysts in our study was a consequence of limitations of the *Col1a1(3.6)*-Cre or the need for additional time for cyst development in the liver. Regardless, the broad expression of *Col1a1(3.6)*-Cre in multiple tissues limits the conclusion that can be drawn from this targeting strategy [32].

Regardless, unlike the embryonic lethality of global *Pkd1* null mice [7], [15], the conditional *Pkd1* null mice are born alive and exhibit a 50% 6-week survival rate, thereby creating a new model to study polycystic kidney and pancreatic cystic disease postnatally. We found that the expression of growth factor such as TGF-β was significantly increased in homozygous *Pkd1^Col1a1(3.6)-^* ^cKO^ kidney at 6 weeks of age, which would stimulate the epithelial cell phenotype's transformation, producing myofibroblasts and secreting extracellular matrix (ECM). In fact, *snail1* and *vimentin*, the EMT markers, and α-SMA and *Col1a1*, the fibrosis markers, were markedly upregulated in homozygous *Pkd1^Col1a1(3.6)-^* ^cKO^ kidney compared with controls at 6 weeks of age. These findings agree with previous reports that polycystic kidney disease triggers the onset of epithelial-mesenchymal transition (EMT) and renal fibrosis [65], [66], [67], [68], [69], [70].

In conclusion, the conditional deletion of *Pkd1* from mesenchymal lineage results in both defective bone formation and polycystic kidney and pancreatic but not liver disease, indicating that *v*al function in mesenchymal precursors to regulate skeletal, renal, and pancreatic development. The long-term survival of *Pkd1^Col1a1(3.6)-^* ^cKO^ mice establishes a potential model to study postnatal interventions to retard cyst formation.

## Materials and Methods

### Mice

We obtained the floxed *Pkd1* mouse (*Pkd1* ^flox/flox^) which harbors two *loxP* sites flanking exon 2–4 of the *Pkd1* gene from Dr. Gregory Germino at Johns Hopkins University [25] and *Col1a1(3.6)*-Cre transgenic mouse, which has activity in mesenchymal precursors, from the University of Missouri-Kansas City [31]. We crossed the floxed heterozygous *Pkd1* ^flox/+^ mice with heterozygous *Col1a1(3.6)*-Cre mice to generate double heterozygous *Col1a1(3.6)*-Cre;*Pkd1* ^flox/+^ mice. Then the resulting *Col1a1(3.6)*-Cre; *Pkd1* ^flox/+^ mice were bred with homozygous *Pkd1* ^flox/flox^ mice to generate conditional *Pkd1* homozygous mice (*Col1a1(3.6* -Cre;*Pkd1* ^flox/flox^), conditional *Pkd1* heterozygous mice (*Col1a1(3.6)*-Cre;*Pkd1* ^flox/+^), homozygous *Pkd1* ^flox/flox^ mice, and heterozygous *Pkd1* ^flox/+^ mice. To achieve greater Cre-mediated reduction in *Pkd1* conditional deletion, we bred double heterozygous *Col1a1(3.6)* -Cre;*Pkd1^null^* ^/+^ mice with homozygous *Pkd1* ^flox/flox^ mice to generate excised floxed *Pkd1* heterozygous (*Col1a1(3.6)* -Cre;*Pkd1* ^flox/+^) and null mice (*Col1a1(3.6)* -Cre;*Pkd1* ^flox/null^), as well as *Pkd1* heterozygous mice (*Pkd1* ^flox/null^) and *Col1a1(3.6)*-Cre negative control mice (*Pkd1* ^flox/+^, equivalent to wild type). These mice were used for phenotypic analysis. Mouse embryos between embryonic day 14.5 (E14.5) and E18.5 were collected from timed pregnant mice. All animal research was conducted according to guidelines provided by the National Institutes of Health and the Institute of Laboratory Animal Resources, National Research Council. The University of Tennessee Health Science Center's Animal Care and Use Committee approved all animal studies (Protocol number: 1885R2).

### Genotyping polymerase chain reaction (PCR) to detect deletions

Genomic DNA was prepared from different tissues using standard procedures, and genotyping PCR was performed to detect *Col1a1(3.6)*-Cre-mediated deletions of the *Pkd1* gene as previously described [17], [18]. In this regard, *Pkd1* wild-type (*Pkd1* ^+^) and floxed (*Pkd1* ^flox^) alleles were identified in 2% agarose gels as 130- and 670-bp bands, respectively. The conditional deleted *Pkd1* (*Pkd1* ^Δflox^) allele was detected as a 0.85-kb band in 1% agarose gels as previously described [17], [18].

### Bone densitometry, histomorphometric, marrow adipocyte staining, and microcomputed tomography analyses

BMD of femurs was assessed at 6 weeks of age with a LUNAR_PIXIMUS_ bone densitometer (Lunar Corp., Madison, WI, USA). Calcein (Sigma-Aldrich, St. Louis, MO, USA) double labeling of bone and histomorphometric analyses of periosteal MAR in tibias were performed using the osteomeasure analysis system (OsteoMetrics, Decatur, GA, USA) [71], [72]. The distal femoral metaphyses were also scanned with a Scanco µCT 40 (Scanco Medical AG, Brüttisellen, Switzerland). 3D-images were analyzed to determine bone volume/trabecular volume and cortical thickness as previously described [71]. For detection of bone marrow adipocytes, whole intact femurs or tibiae with encapsulated marrow were dissected from 6-week-old mice, fixed for 48 h in phosphate-buffered paraformaldehyde, decalcified in 14% EDTA, and then stained with aqueous osmium tetroxide (OsO4) for quantification of fat volume, density, and distribution by μCT analysis [17]. In addition, the cryosectioning was performed for Oil Red O lipid staining as previously reported in our laboratory [17].

### Tissue histology and kidney fibrosis staining

The kidneys, livers, and pancreases from E14.5-, E15.5-, E16.5-, E18.5-, newborn (P0)-, postnatal day 3 (P3)-, 7 (P7)-, 14(P14)-, 4-, 6-, and 8-week-old mice were collected and fixed in 4% paraformaldehyde for 24 h and then embedded in paraffin. Eight-micrometer tissue sections were used for hematoxylin-eosin staining as described previously [17]. Masson-Trichrome staining for collagen fibers on kidney sections was also performed according to the manufacturer's instructions (Polysciences Inc., Warrington PA, USA).

### Serum biochemistry

Serum osteocalcin levels were measured using a mouse Osteocalcin EIA kit (Biomedical Technologies, Inc., Stoughton, MA, USA). Serum BUN was determined using a BUN diagnostic kit from Pointe Scientific, Inc (Canton, MI, USA). Serum calcium (Ca) was measured by the colorimetric cresolphthalein binding method, and phosphorus (P) was measured by the phosphomolybdate –ascorbic acid method (Stanbio Laboratory, Boerne, TX, USA). Serum osteoprotegerin (OPG) and Rank ligand (RankL) were measured using mouse ELISA kits (Quantikine^®^, R&D Systems, Minneapolis, MN, USA), and serum tartrate-resistant acid phosphatase (TRAP) was assayed with the ELISA-based SBA Sciences mouse TRAP^TM^ assay (Immunodiagnostic Systems, Fountain Hills, AZ, USA). Serum parathyroid hormone (PTH) levels were measured using the Mouse Intact PTH ELISA kit (Immutopics, Carlsbad, CA, USA). Serum FGF23 levels were measured using the FGF23 ELISA kit (Kainos Laboratories, Tokyo, Japan).

### Bone RNA isolation and real-time reverse transcriptase (RT)-PCR

For quantitative real-time RT-PCR, 1.0 μg total RNA isolated from calvaria, kidney, liver, and whole tibias of 6-week-old control and *Col1a1(3.6)*-Cre-mediated *Pkd1*-deficient mice was reverse transcribed as previously described [34]. PCR reactions contained 20 ηg template (cDNA or RNA), 375 ηM each forward and reverse primers, and 1X SsoFast^TM^ EvaGreen® supermix (Bio-Rad, Hercules, CA, USA) in a total of 10 μl reaction volume. The threshold cycle (Ct) of tested gene product from the indicated genotype was normalized to the Ct for cyclophilin A. Expression of total *Pkd1* transcripts was performed using the following *Pkd1-*allele-specific primers in exons 2–4: forward primer of normal *Pkd1* ^+^ transcript (*Pkd1* ^+^ plus *Pkd1* ^flox^): 5′-ATA GGG CTC CTG GTG AAC CT-3′, and reverse primer: 5′-CCA CAG TTG CAC TCA AAT GG-3′. The normal *Pkd1* ^+^ vs. cyclophilin A was normalized to the mean ratio of five control mice, which was set to 1. The percentage of conditional deleted *Pkd1*(*Pkd1* ^Δflox^) transcripts was calculated from the relative levels of the normal *Pkd1* ^+^ transcripts in different *Pkd1*-deficient mice [73]. All primer information of other genes used in real-time RT-PCR can be found in our previous report [18].

### Cell proliferation, osteoblastic differentiation, and gene expression profiles in immortalized osteoblast cultures

Calvaria from E17.5 control and *Pkd1*-deficient embryos were used to isolate primary osteoblasts by sequential collagenase digestion at 37°C. To engineer immortal osteoblast cell lines, isolated primary osteoblasts were infected using a retroviral vector carrying SV40 large and small T antigen as previously described [34], [74]. Briefly, cells were grown in 100-mm plates at 50–60% confluence the day before infection. On the day of infection, the medium was removed and replaced with medium containing SV40 large and small T antigen-helper-free viral supernatant in the presence of 4 mg/ml of polybrene (Sigma, St. Louis, MO, USA) for 48 h. The cells were allowed to recover for 72 h followed by selection with 1 mg/ml puromycin (Sigma) for up to 15 days. The immortalized osteoblasts were cultured in α-MEM containing 10% FBS and 1% penicillin and streptomycin (P/S) and characterized following the protocols below. Cell proliferation was detected by BrdU incorporation assays following the manufacturer's directions (QIA58, Calbiochem, Gibbstown, NJ, USA). To induce differentiation, the immortalized osteoblasts were plated at a density of 2×10^4^ cells per well in a 12-well plate and 4×10^4^ cells per well in a 6-well plate and grown up to 21 days in α-MEM containing 10% FBS supplemented with 5 mM β-glycerophosphate and 25 µg/ml ascorbic acid. ALP activity and Alizarin red-S histochemical staining for mineralization were performed as previously described [16], [34]. Total DNA content was measured with a PicoGreen^®^ dsDNA quantitation reagent and kit (Molecular Probes, Eugene, OR, USA). Protein concentrations of the supernatant were determined with a Bradford protein assay kit (Bio-Rad, Heumrcules, CA, USA). For gene expression profiles, 1.0 μg of total RNA was isolated from primary osteoblasts cultured 4, 12, and 21 days in differentiation media. The cDNAs were generated using an iScript reverse transcription kit (Bio-Rad). PCR reactions contained 20 ηg template (cRNA or cDNA), 375 ηmol each forward and reverse primers, 1X SsoFast^TM^ EvaGreen® supermix (Bio-Rad) in a total of 10 µl reaction volume. The Ct of tested gene product from the indicated genotype was normalized to the Ct for cyclophilin A as previously described [16], [34], [36].

### Statistical analysis

We evaluated differences between two groups by unpaired t-test and multiple groups by one-way analysis of variance. All values are expressed as means ± SD. All computations were performed using GraphPad Prism5 (GraphPad Software Inc. La Jolla, CA, USA).
